# A Central Role for C1q/TNF-Related Protein 13 (CTRP13) in Modulating Food Intake and Body Weight

**DOI:** 10.1371/journal.pone.0062862

**Published:** 2013-04-25

**Authors:** Mardi S. Byerly, Roy Swanson, Zhikui Wei, Marcus M. Seldin, Patrick S. McCulloh, G. William Wong

**Affiliations:** 1 Department of Physiology, Johns Hopkins University School of Medicine, Baltimore, Maryland, United States of America; 2 Center for Metabolism and Obesity Research, Johns Hopkins University School of Medicine, Baltimore, Maryland, United States of America; University of Maryland School of Pharmacy, United States of America

## Abstract

C1q/TNF-related protein 13 (CTRP13), a hormone secreted by adipose tissue (adipokines), helps regulate glucose metabolism in peripheral tissues. We previously reported that *CTRP13* expression is increased in obese and hyperphagic leptin-deficient mice, suggesting that it may modulate food intake and body weight. CTRP13 is also expressed in the brain, although its role in modulating whole-body energy balance remains unknown. Here, we show that CTRP13 is a novel anorexigenic factor in the mouse brain. Quantitative PCR demonstrated that food restriction downregulates *Ctrp13* expression in mouse hypothalamus, while high-fat feeding upregulates expression. Central administration of recombinant CTRP13 suppressed food intake and reduced body weight in mice. Further, CTRP13 and the orexigenic neuropeptide agouti-related protein (AgRP) reciprocally regulate each other’s expression in the hypothalamus: central delivery of CTRP13 suppressed *Agrp* expression, while delivery of AgRP increased *Ctrp13* expression. Food restriction alone reduced *Ctrp13* and increased orexigenic neuropeptide gene (*Npy* and *Agrp*) expression in the hypothalamus; in contrast, when food restriction was coupled to enhanced physical activity in an activity-based anorexia (ABA) mouse model, hypothalamic expression of both *Ctrp13* and *Agrp* were upregulated. Taken together, these results suggest that CTRP13 and AgRP form a hypothalamic feedback loop to modulate food intake and that this neural circuit may be disrupted in an anorexic-like condition.

## Introduction

The hypothalamus plays a central role in integrating hormonal and neural signaling pathways to modulate food intake and body weight [1]. Neurons in the arcuate nucleus of the hypothalamus are particularly important to this process. For example, neurons in the arcuate nucleus respond to leptin, a hormone secreted by adipocytes (known as an adipokine) in proportion to fat mass, to suppress food intake [2]. Different populations of neurons in the arcuate nucleus variably influence behavior: activation of neuropeptide Y/agouti-related protein (NPY/AgRP)-expressing neurons promote food intake; activation of pro-opiomelanocortin/cocaine- and amphetamine-regulated transcript (POMC/CART)-expressing neurons suppress food intake [1]. Interestingly, individuals diagnosed with the eating disorder anorexia nervosa (AN) have increased circulating levels of AgRP [3], [4], and a polymorphism in the coding region of *AgRP* is associated with AN [5]. Further, leptin and another widely-studied adipokine, adiponectin, act in the hypothalamus to regulate body weight [6], [7], [8]. Indeed, plasma leptin levels decrease in an anorexic state [9], while circulating levels of adiponectin vary depending on the type of AN [10], [11], [12], [13].

We have recently identified and characterized a novel and conserved family of secreted hormones, the C1q/TNF-related proteins (CTRP1-15) [14], [15], [16], [17], [18], [19], [20]. Each CTRP has a unique tissue expression profile, and several have been shown to play important and distinct roles in regulating glucose and fatty acid metabolism [17], [18], [19], [21], [22]. Adipose tissues and the brain predominantly express CTRP13, and circulating levels of CTRP13 are increased in leptin-deficient obese (*ob/ob*) hyperphagic mice [18]. Recombinant CTRP13 promotes glucose uptake in cultured myotubes, hepatocytes, and adipocytes and ameliorates fatty acid-induced insulin resistance in cultured hepatocytes by suppressing lipid-induced stress signaling (i.e., JNK) [18], suggesting that increases in CTRP13 *in vivo* may be a compensatory response to hyperglycemia. While the action of CTRP13 on peripheral tissues has been demonstrated, its potential role in regulating metabolism in the central nervous system (CNS) remains unknown.

Here, we provide evidence that CTRP13 acts in the brain as an anorexigenic signal to modulate food intake and body weight. Our *in vivo* results suggest that CTRP13 and AgRP form a neural feedback loop to modulate food intake and that this feedback mechanism is disrupted in an activity-based anorexia (ABA) mouse model. These results have implications for CTPR13 in understanding AN, a complex but poorly understood eating disorder in humans.

## Materials and Methods

### Animals

Wild-type (WT) male C57BL/6J mice (6–8 weeks old, The Jackson Laboratories, Bar Harbor, ME) were used for these studies. The neural circuitry of the hypothalamus has been largely investigated in male mice. The primary goal of this study was to characterize the central effects of CTRP13 on food intake and peripheral energy metabolism. We chose to utilize male mice in our current study so that we can better relate our results to prior published studies with regard to alterations in neural circuitry induced by central administration of recombinant CTRP13. Mice were given *ad libitum* access to water and standard rodent laboratory chow (2018, Harland-Teklad) and housed under a 12∶12 hour dark:light cycle. Diet-induced obese (DIO) male C57BL/6 mice (The Jackson Laboratories) were fed a 60% high-fat diet (HFD; Research Diets; D12492) for 8 weeks after weaning. Age-matched chow-fed controls were also purchased from The Jackson Laboratories. The studies were conducted using WT male mice fed a standard rodent laboratory chow, except for the neuropeptide analysis, which utilized DIO male mice. All studies accorded with the recommendations in the Guide for the Care and Use of Laboratory Animals of the National Institutes of Health. All animal experiments were approved by the Animal Care and Use Committee of The Johns Hopkins University School of Medicine (protocol number MO11M49).

### Activity-based Anorexia Paradigm and Metabolic Measurements

Animals were individually housed in 40 cm×40 cm×30 cm clear plexiglass cages, which were used to measure all metabolic parameters. Animals received four days for adaptation to individual housing prior to the start of the experiment. CLAMS (Comprehensive Laboratory Animal Monitoring System) (Columbus Instruments, Columbus, OH) was used to measure body weight, whole-body metabolism [VO_2_, VCO_2_, respiratory exchange ratio (RER), and energy expenditure (EE)], ambulatory activity, running wheel activity, water consumption, and food intake. We adapted the rat ABA paradigm [23], [24], [25] to our present mouse study. As a control, the *ad libitum* group (AL) was provided access to standard laboratory chow with no running wheel. The running wheel (RW) group was provided *ad libitum* access to standard laboratory chow and free access to a running wheel. The food restricted (FR) group was allowed 2 h of access to standard laboratory chow each day at the beginning of the dark cycle (days 1–5), with no access to a running wheel. The ABA group was allowed 2 h of access to standard laboratory chow each day at the beginning of the dark cycle (days 1–5), as well as free access to a running wheel. All CLAMS data presented represent the average 24-h value collected on days 4 and 5. Sera were collected from tail bleeds on day 5 for immunoblot analysis of CTRP13. Later, mice from the FR and ABA groups were allowed 4 h of access to standard laboratory chow each day at the beginning of the dark cycle for an additional 9 days (days 6–14). The body weight, food intake, and metabolic data presented in the results section were collected during the first week of exposure to the ABA paradigm, and metabolic data collected after the first seven days are not shown. After the second week of exposure to the ABA paradigm, hypothalami were then collected for real-time PCR analysis of *Ctrp13* and neuropeptide gene expression.

### Recombinant Protein Purification

Full-length recombinant CTRP13 with a C-terminal FLAG epitope tag (DYKDDDDK) was expressed in HEK 293 cells (GripTite™ 293 cell line from Invitrogen, Carlsbad, CA). Expression of recombinant protein in mammalian cells was critically important to ensure that all proper posttranslational modifications of CTRP13 and its assembly into a higher-order oligomeric form were preserved [18]. Serum-free conditioned media (Opti-MEM, Invitrogen) containing the secreted CTRP13 was purified as previously described [18]. Purified proteins were dialyzed against 20 mM Hepes buffer (pH 8.0) containing 135 mM NaCl in a 10 kDa cut-off Slide-A-Lyzer (Thermo Fisher Scientific, Waltham, MA). Protein concentration was determined using a Coomassie Plus protein assay reagent (Thermo Fisher Scientific, Rockville, MD), and samples were stored at −80°C.

### Stereotaxic Cannulation and Recombinant Protein Injection

A unilateral cannula was implanted into the lateral ventricle of the brain as previously described [26]. Only wild-type C57BL/6 male mice were cannulated. None of the ABA mice were cannulated or injected with recombinant protein. The correct placement of the cannula was verified by intracerebroventricular (ICV) injection of 2 µL of NPY (a total of 2 nmol was administered per mouse) (American Peptide Company, Sunnyvale, CA). A 1-h food intake test was given during the light cycle, with an approximate 40% increase in food intake relative to saline injection considered successful. Prior to recombinant protein injection, mice were individually housed in indirect calorimetry metabolic chambers (CLAMS) to allow adaptation for 4 days. Two µL of recombinant protein (150 ng/µL) were ICV injected at the beginning of the dark cycle and body weight, food intake, and physical activity was measured over a 24-h period after protein injection. Vehicle [20 mM Hepes (pH 8.0) containing 135 mM NaCl] was used as control in all ICV injection experiments.

### RNA Extraction and Quantitative Real-time PCR

Using the anterior commisure and the oculomotor nerve as neuroanatomical markers, adult mouse hypothalami were dissected in phosphate buffered saline solution. Total RNA was extracted using the RNeasy Midi kit (Qiagen, Valencia, CA). cDNA was generated using SuperScript® II Reverse Transcriptase (Invitrogen) and random hexamers from 1 µg of RNA. The default PCR protocol was performed on an Applied Biosystems Prism 7500 Sequence Detection System using SyBR® Green PCR Master Mix (Applied Biosystems, Carlsbad, CA). The qPCR output provided a Ct value for the threshold cycle and the ΔCt value was obtained by normalizing the data to *18S rRNA*. The ΔΔCt value (relative expression level) was generated by normalizing to the average control value. Primers used included: *Nenf* forward 5′-TCTGTAGCCGCTCTATCTGG-3′ and reverse 5′-GTGTCGTG AGTGAGGTCTGC-3′; *AgRP* forward 5′-ATGCTAGGTAACAAGCGAATGG-3′ and reverse 5′-CAGACTTAGACCTGGGAACTCT-3′; *Npy* forward 5′-CTAGGTAACAAGCGAATGG-3′ and reverse 5′-TGTCGCAGAGCGGAGTAGTAT-3′; *18 S rRNA* forward 5′-GCAATTATTCC CCATGAACG-3′ and reverse 5′-GGCCTCACTAAACCATCCAA-3′.

### Immunoblot Analysis

Rabbit polyclonal anti-CTRP13 antibody directed against the C-terminal globular domain was generated as previously described [18]. One µL-equivalent serum samples were suspended in NuPAGE LDS sample buffer and heated for 5 minutes at 90°C. Samples were electrophoresed on 10% BisTris NuPAGE gels (Invitrogen, Carlsbad, CA), transferred to 0.2 µm Protran BA83 nitrocellulose membranes (GE Healthcare, Piscataway, NJ), and probed with the CTRP13 primary antibody and appropriate HRP-conjugated secondary antibody. Bands were visualized with Immobilon Western HRP substrate peroxide solution (Millipore, Billerica, MA), captured with MultiImage III FluorChem® Q (Alpha Innotech, San Leandro, CA), and quantified using Alphaview Software (Alpha Innotech).

### Statistical Analysis

All value comparisons were made using either a *t*-test, one-way ANOVA or repeated measures ANOVA (Statistica v.8.0, Tulsa, OK). Values reported are means±SEM. *P*<0.05 was considered statistically significant.

## Results

### Diet-induced Obesity Affects CTRP13 Transcript Levels in the Hypothalamus

**Figure 1:**
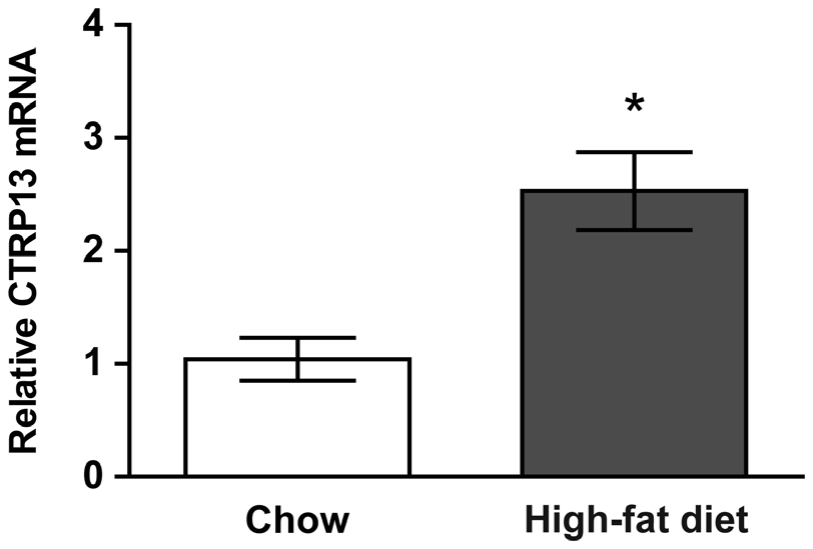
Hypothalamic *CTRP13* expression increased in mice fed a high-fat diet (HFD). Food was removed for 2 h before hypothalami were harvested from mice fed an HFD (n = 5) or from mice fed a standard chow diet (n = 4). mRNA levels were measured using quantitative real-time PCR. Data are means ± SEM,*P<0.05.

In our previous study, the expression levels of *Ctrp13* were measured only in the periphery [18]. Here, *Ctrp13* transcript levels were measured in the CNS, specifically the hypothalamus, under metabolic stress induced by high-fat feeding in DIO mice. As measured by quantitative real-time PCR, *Ctrp13* expression in the hypothalamus increased in mice fed a HFD relative to mice fed a standard chow diet (Figure 1). Given the importance of the hypothalamus in controlling food intake and peripheral energy metabolism [1], this result suggests that hypothalamic expression of *Ctrp13* may be physiologically relevant in the context of food intake and body weight regulation.

### Central Administration of Recombinant CTRP13 Suppresses Food Intake and Body Weight

**Figure 2:**
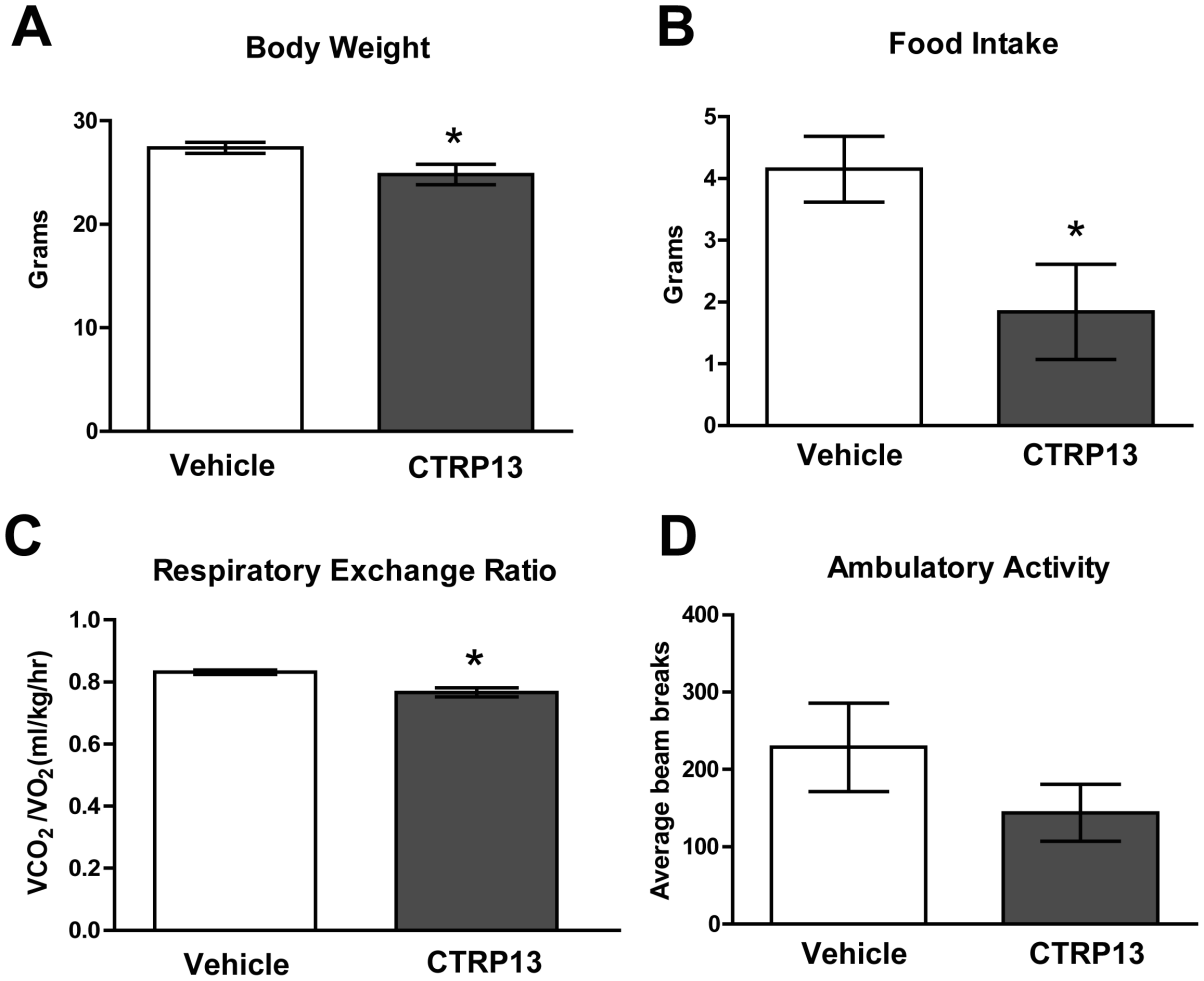
Central administration of recombinant CTRP13 suppressed food intake and reduced body weight. A) Body weight, B) food intake, and C) respiratory exchange ratio (RER) were measured by CLAMS 24 h after ICV injection of recombinant CTRP13 (n = 8) or vehicle control (n = 7). D) Ambulatory activity levels over a 20-h period were also measured after ICV injection of CTRP13 (n = 8) relative to vehicle-injected (n = 7) mice. Data are means ± SEM,*P<0.05.

To address whether the upregulated expression of hypothalamic *CTRP13* in HFD-fed mice represents a possible compensatory response to an altered state of energy balance, we injected purified recombinant CTRP13 or vehicle control into the lateral ventricle of chow-fed mice to determine its potential appetite-regulating function in the brain. ICV administration of a single dose of recombinant CTRP13 (2 µL of 150 ng/µL) significantly decreased body weight (8%) (Figure 2A) and suppressed food intake (56%) (Figure 2B) over a 24-h period, relative to vehicle-injected controls. RER also significantly decreased (8%) in CTRP13-administered mice relative to vehicle-injected controls (Figure 2C). Previous studies have shown that a 0.10 ratio reduction in RER reduces O_2_ consumption by carbohydrate oxidation by ∼34% and increases O_2_ consumption by fat oxidation by ∼34% [27]. CTRP13-induced suppression of food intake could result in greater fatty acid release from adipocytes, supplying substrates for fat oxidation in the liver and skeletal muscle and possibly accounting for the decreased RER. Importantly, there was no significant change in physical activity levels (over a 20-h period) between the CTRP13- and vehicle-injected groups (Figure 2D), suggesting that the central effect of CTRP13 in appetite suppression (over a similar time period) was not due to non-specific physical illness induced by central delivery of the recombinant protein.

### Establishment of an Activity-based Anorexia (ABA) Mouse Model

Severe restriction of food intake is characteristic of AN, and hyperactivity is common among these individuals [28], [29]. Animal models of ABA mimic core features of AN in humans, including food restriction, reduced body weight, and enhanced physical activity [24], [25], [30]. We adapted the rat ABA paradigms [23], [24], [25] to our present mouse study. Variations in ABA paradigm or mouse strain or sex can affect food intake and body weight loss [31], [32], [33], [34], [35], [36]. Development of ABA in mice can also be modulated by the length of time that food intake is permitted over a 24-h period (e.g., 1, 2, 4, or 6 h), and the time at which food is delivered for consumption (e.g., light or dark cycle) [31], [32], [33], [34], [35], [36]. In the present study, mice were maintained on the ABA paradigm for a longer period of time to validate the paradigm and to characterize alterations in neural circuitry due to long-term exposure to ABA (i.e., two weeks rather than one).

**Figure 3:**
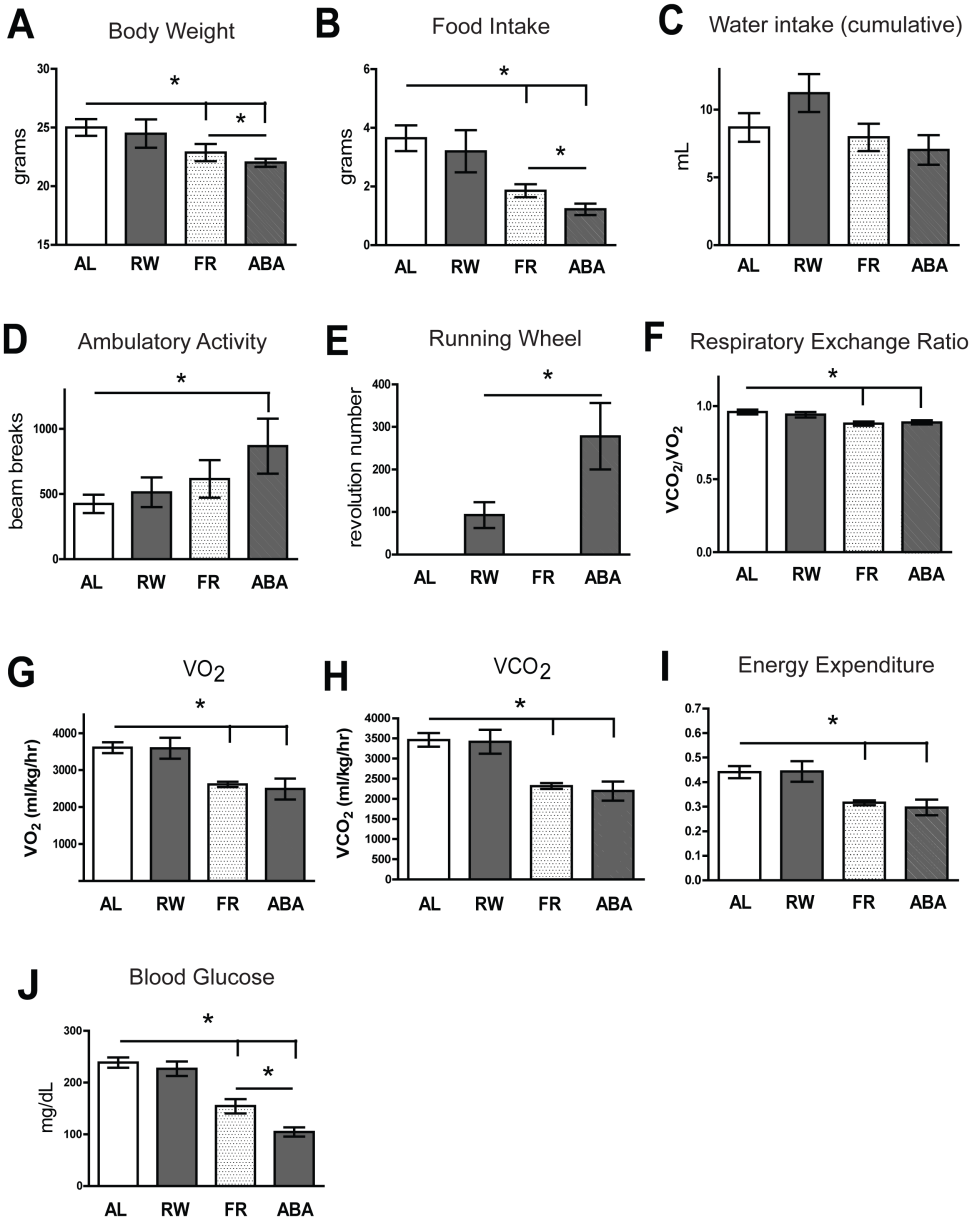
Metabolic parameters and behavior associated with activity-based anorexia (ABA) paradigm. A) Body weight, B) food intake, C) water intake, D) ambulatory activity, E) running wheel activity, F) respiratory exchange ratio (RER), G) VO_2_, H) VCO_2_, I) Heat/energy expenditure, and J) blood glucose were measured in AL (n = 5), RW (n = 5), FR (n = 6) and ABA (n = 5) groups by CLAMS. Values represent means ± SEM, *P<0.05 vs. AL, averaged over paradigm exposure days 4 and 5, and day 5 for blood glucose values. (AL = *ad libitum* food access; RW = *ad libitum* food access and free access to a running wheel; FR = 2-h food access per day; ABA = 2-h food access per day and free access to a running wheel).

**Figure 4:**
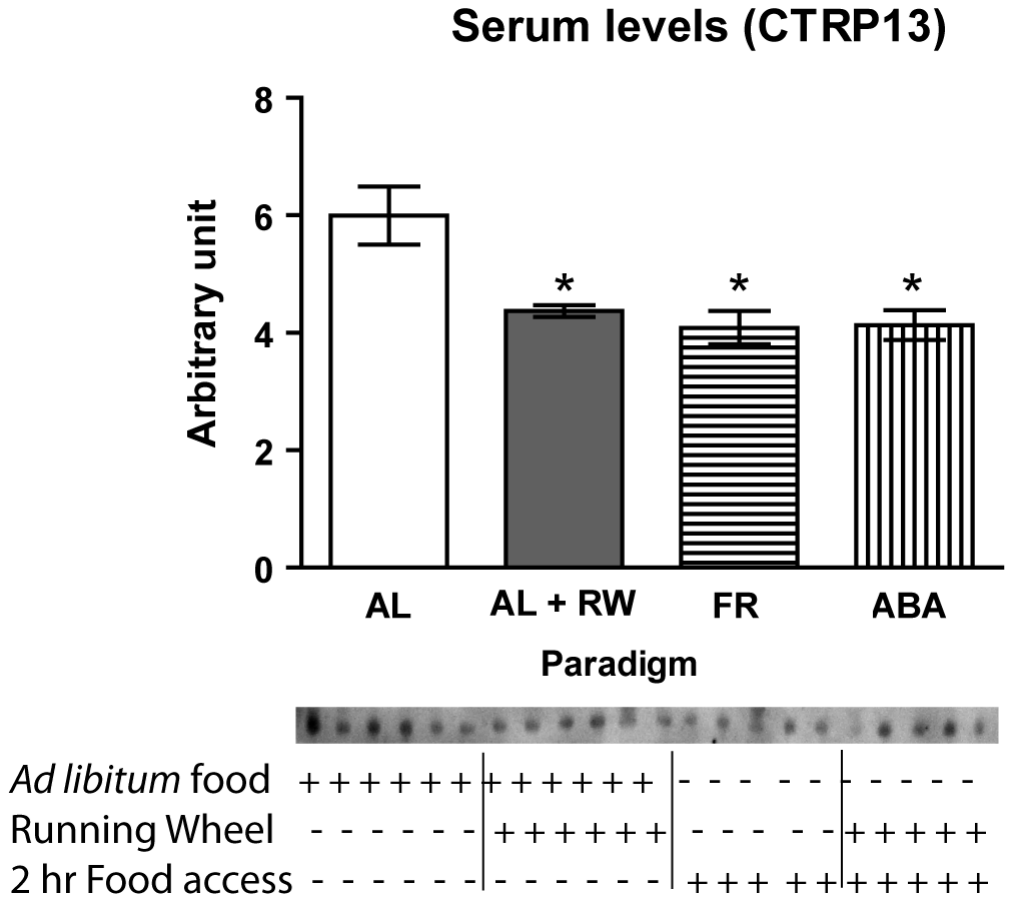
Metabolic parameters and behavior associated with activity-based anorexia (ABA) paradigm.Activity-based anorexia (ABA) paradigm reduced circulating levels of CTRP13. Immunoblot of serum levels of CTRP13 in AL (n = 5), RW (n = 5), FR (n = 6), and ABA (n = 5) groups were quantified. CTRP13 serum levels were decreased in the RW, FR, and ABA groups relative to the AL group. Values shown are means ± SEM, *P<0.05 vs. AL. (AL = *ad libitum* food access; RW = *ad libitum* food access and free access to a running wheel; FR = 2-h food access per day; ABA = 2-h food access per day and free access to a running wheel).

After being exposed to the ABA paradigm group variables for five days, the FR and ABA groups were allowed 2 h access to food per day. Mice in these two groups demonstrated decreased body weight (Figure 3A), decreased food intake (Figure 3B), no change in cumulative water intake (Figure 3C), increased ambulatory activity (Figure 3D), decreased RER (Figure 3F), decreased basal metabolic rate (VO_2_ and VCO_2_) (Figure 3G and H), decreased heat/energy expenditure (Figure 3I), and decreased blood glucose (Figure 3J) relative to the AL group. The ABA group demonstrated increased running wheel activity relative to the RW group (Figure 3E). No differences were observed between the FR and ABA groups on the first two days of the paradigm (data not shown). However, from day three we observed differences in body weight, food intake, and blood glucose between the FR and ABA groups (Figures 3A, B, and J, respectively). These data indicated that we established an anorexia-like mouse model (decreased food intake and increased physical activity) by restricting food intake while giving the mice free access to a running wheel. Western blotting of sera harvested from these 4 groups of mice showed that circulating levels of CTRP13 in the periphery were decreased (∼30%) in the ABA, FR, and RW groups relative to the AL group (Figure 4). Decreased food intake and body weight were observed in both the ABA and FR groups, but not the RW group, compared to AL, despite decreased serum levels of CTRP13 in all three groups. This suggests that other factors in addition to food intake and body weight may influence peripheral CTRP13 levels.

### CTRP13 administration In the Mouse CNS Demonstrates a Phenotype Resembling ABA

**Figure 5:**
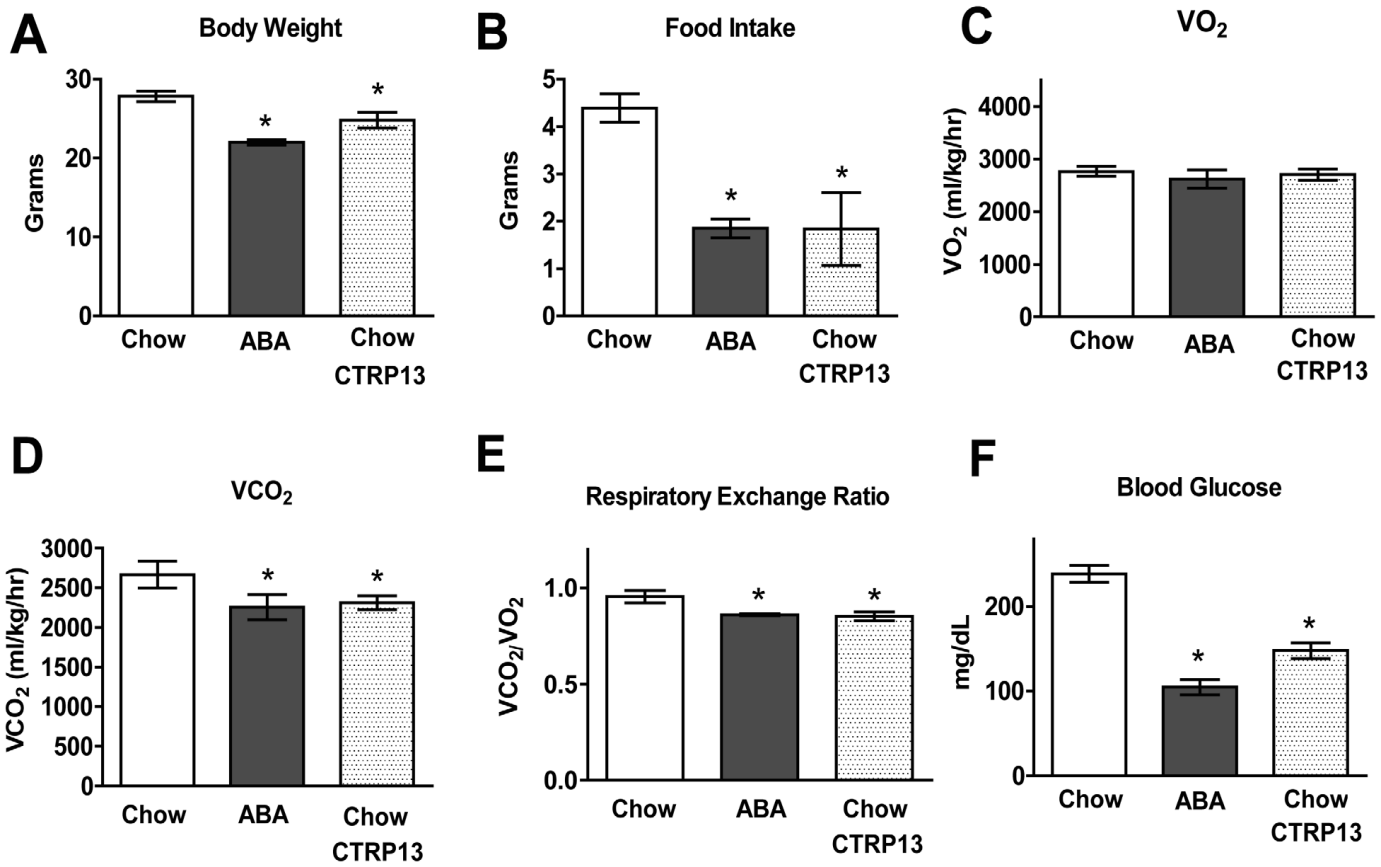
Comparison of behavior and metabolic profiles of mice ICV-injected with vehicle or CTRP13 and mice subjected to the activity-based anorexia (ABA) paradigm. A) Body weight, B) food intake, C) VO_2_, D) VCO_2_, E) respiratory exchange ratio (RER), and F) blood glucose were measured by CLAMS in ABA (n = 5), CTRP13-injected (n = 8), and vehicle-injected control (n = 7) mice. Data are means ± SEM,*P<0.05.

We conducted a side-by-side comparison between non-cannulated ABA mice, chow-fed mice injected centrally with vehicle control, and chow-fed mice injected centrally with a single dose of recombinant CTRP13 (2 µL of 150 ng/µl) by assessing whole-body metabolic parameters and measuring random blood glucose levels. A lower RER is indicative of a greater reliance on fatty acids as substrates for energy production and is expected when mice have a suppressed food intake. ICV delivery of recombinant CTRP13 significantly reduced body weight (Figure 5A), food intake (Figure 5B), VCO_2_ (Figure 5 D), RER (Figure 5E), and blood glucose levels (Figure 5F) relative to vehicle-injected controls, without affecting VO_2_ (Figure 5C). Similarly, ABA mice also demonstrated decreases in all the same metabolic parameters. Together, these data support a satiety role of CTRP13 in the CNS.

### Altered Expression of *CTRP13* and Orexigenic Neuropeptide Genes in the Hypothalamus of ABA Mice

**Figure 6:**
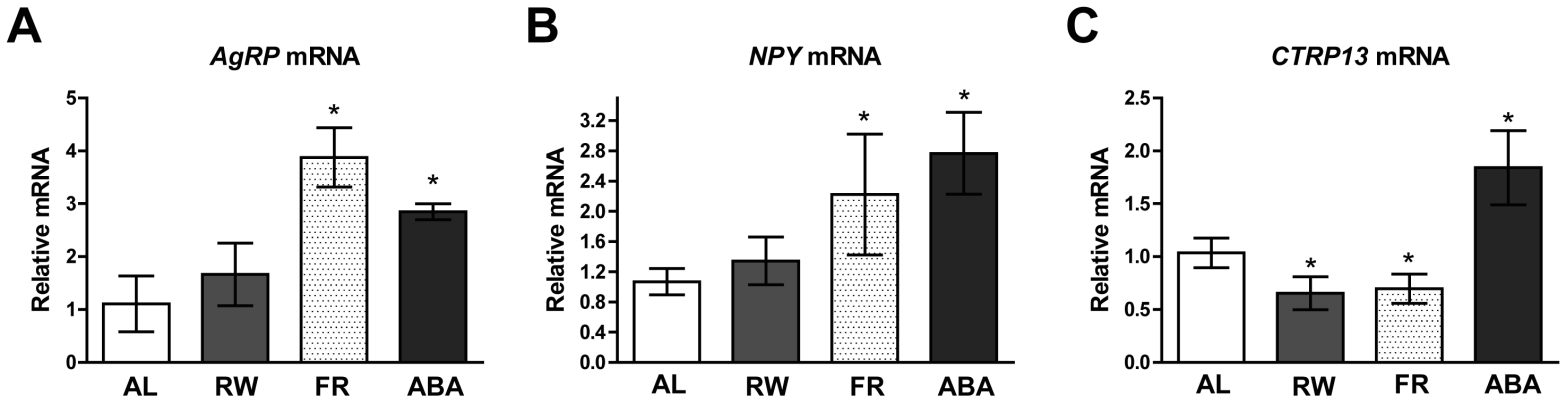
Activity-based anorexia (ABA) paradigm altered hypothalamic *Ctrp13* *Ctrp13* and neuropeptide gene expression. Quantitative real-time PCR measured A) *Agrp* B) *Ctrp13*, and C) *Npy* mRNA levels in the hypothalamic of AL (n = 5), RW (n = 5), FR (n = 6), ABA (n = 5) and AL (n = 5) mice. Data are means ± SEM,*P<0.05 vs. vehicle. (AL = *ad libitum* food access; RW = *ad libitum* food access and free access to a running wheel; FR = 2-h food access per day; ABA = 2-h food access per day and free access to a running wheel).

Given the important role of orexigenic neuropeptides (NPY and AgRP) in the hypothalamus to modulate ingestive behavior [1], we assessed their expression levels and that of *CTRP13* in the 4 groups of mice after two weeks of exposure to AL (*ad libitum*), RW (*ad libitum* with running wheel), FR (food restriction), or ABA (food restriction with running wheel) paradigms. Under the condition of negative energy balance (e.g., fasted or food-restricted mice), *Agrp* expression levels in the hypothalamus increase to stimulate food consumption [1]. Real-time PCR detected significantly increased *Npy and Agrp* expression in the hypothalamus of food-restricted FR and ABA groups relative to the AL group (Figure 6A–B). Both FR and RW groups had significantly decreased *Ctrp13* expression in the hypothalamus relative to the AL group (Figure 6C). This result was consistent with decreased CTRP13 serum levels observed for both the FR and RW groups (Figure 4), as expected given the satiety effect of CTRP13 (Figure 2 and 5). However, hypothalamic *Ctrp13* expression in ABA mice significantly increased relative to the AL group, despite comparable reductions in body weight in both FR and ABA groups due to food restriction and decreased serum CTRP13 levels. Persistently upregulated *Ctrp13* expression in food-restricted ABA mice, despite enhanced *Agrp* expression, suggests that the appetite-regulatory neural circuit involving CTPR13 may be dysregulated in the anorexic-like condition and that this may be independent of circulating levels in plasma.

### Reciprocal Regulation of *AgRP* and *CTRP13* Expression in the Hypothalamus

**Figure 7:**
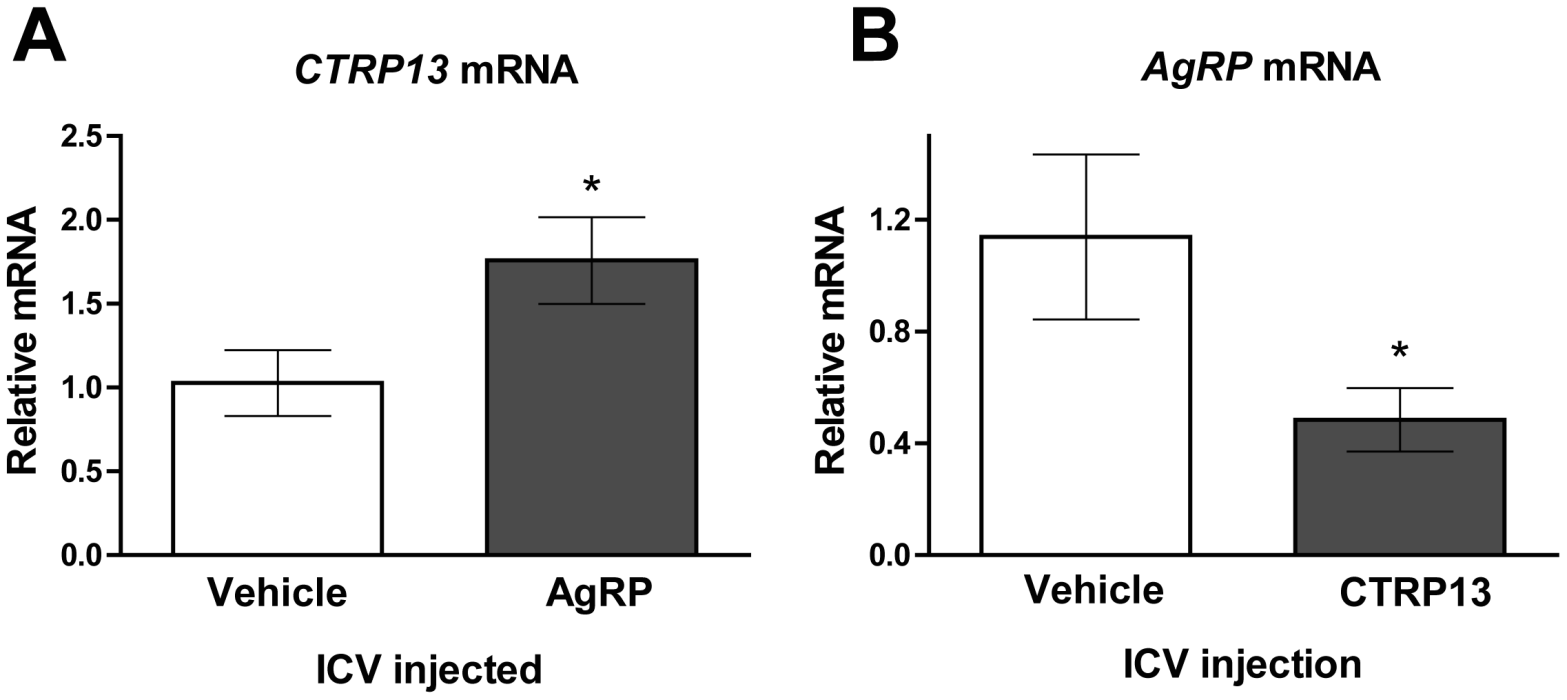
Reciprocal regulation of *Ctrp13* and *Agrp* *Agrp* expression in the hypothalamus. A) Quantitative real-time PCR was used to measure *Ctrp13* expression in mice hypothalami after ICV injection of AgRP (n = 6) or vehicle control (n = 7). B) Quantitative real-time PCR was used to measure *Agrp* expression in mice hypothalami after ICV injection of CTRP13 (n = 6) or vehicle control (n = 7). Data are means ± SEM,*P<0.05.

AgRP promotes food intake, whereas CTRP13, when delivered centrally, suppresses food intake. We asked whether AgRP regulates the hypothalamic expression of *Ctrp13* and vice versa. Recombinant AgRP was delivered into the lateral ventricle of cannulated mice via ICV injection. Following injection, mice were allowed to consume food for 2 h and then were removed from food for 2 h, after which hypothalami were harvested for quantitative real-time PCR analysis. Central administration of the orexigenic neuropeptide AgRP significantly elevated *Ctrp13* transcript levels in the hypothalamus relative to vehicle-injected controls (Figure 7A). Alternatively, recombinant CTRP13 was injected into the lateral ventricle of cannulated mice and hypothalami were collected 3 h after injection. Recombinant CTRP13 significantly reduced *Agrp* expression in the hypothalamus relative to vehicle-injected controls (Figure 7B). These data suggest that the satiety effect of CTRP13 in the brain was due to its ability to acutely reduce expression of orexigenic neuropeptide AgRP, which promotes food intake. Thus, AgRP and CTRP13 reciprocally regulate expression of one another in the hypothalamus; together, these results suggest that CTRP13 and AgRP form a hypothalamic feedback loop to modulate food intake and energy balance and that this feedback circuit appears to be disrupted in the anorexic-like mouse model.

## Discussion

Eating disorders–which include AN, bulimia nervosa, and binge-eating disorder–are serious public health problems, although the molecular underpinnings of these disorders remain largely unknown [37], [38]. High heritability rates and complex patterns of polygenic inheritance underscore the genetic determinants of these complex behavioral disorders [39], [40]. Although distortions of body image are prevalent among individuals with AN [41], central and peripheral factors may also initiate the development of this disease [9], [13], [37], [38].

Here, we provide the first *in vivo* evidence that CTRP13 functions as a novel anorexigenic factor in the hypothalamus to modulate food intake and body weight. Hypothalamic *Ctrp13* expression is dynamically regulated by energy state: 2-week food restriction suppressed expression, but excessive caloric intake from high-fat feeding and exposure to the ABA paradigm enhanced *Ctrp13* expression. Central administration of recombinant CTRP13 suppressed food intake and reduced body weight, in part by decreasing hypothalamic expression of the orexigenic neuropeptide AgRP. In addition, CTRP13 and AgRP regulate one another: central administration of AgRP enhanced *Ctrp13* expression in the hypothalamus, while central administration of CTRP13 suppressed *Agrp* expression. Thus, when AgRP upregulates hypothalamic CTRP13 expression, it may, in turn, act to limit AgRP-mediated food intake. Together, these results suggest that CTRP13 and AgRP form a feedback mechanism to modulate whole-body energy balance.

Patients diagnosed with AN demonstrate a 30–80% increase in physical activity levels [28], [29]. The ABA paradigm used in this study mimics the food restriction and enhanced physical activity observed in anorexic individuals. Patients with AN have decreased body weight, decreased circulating leptin levels, and increased AgRP levels [4]. Likewise, both rats and mice exposed to the ABA paradigm have decreased body weight, decreased plasma leptin levels, and increased hypothalamic AgRP levels [23], [24], [25], [33]. Human and mouse CTRP13 share 96% amino acid identity at the presumed functional C1q domain and share tissue expression profiles (predominantly adipose tissue and the brain) [18]. While this study demonstrates CTRP13’s function in the mouse CNS, it is likely that CTRP13 also plays a similar role in the human CNS to modulate food intake and body weight. Thus, our study provides a rationale for further examination of *CTRP13* expression and function in individuals with AN.

Using the ABA mouse model, we show that *Agrp* and *Ctrp13* mRNA levels are simultaneously elevated in the hypothalamus. During a state of negative energy balance, such as in FR mice, *Agrp* expression increased and *Ctrp13* expression decreased, in the hypothalamus, consistent with their respective roles as orexigenic and anorexigenic factors. In contrast, *Agrp* and *Ctrp13* expression both increased in the hypothalamus of ABA mice, despite an equal degree of food restriction in both ABA and FR mice. These observations suggest that the potential hypothalamic AgRP-CTRP13 feedback loop is dysregulated in ABA mice. We speculate that in an anorexic-like state, the hypothalamic neurons may not appropriately respond to signals to alter *Ctrp13* expression. Therefore, persistently high expression of both orexigenic (*AgRP*) and anorexigenic (*CTRP13*) genes in an anorexic-like state may be causally linked to the pathogenesis of AN.
